# Supplementary material for: CRISPR/Cas9 recombineering‐mediated deep mutational scanning of essential genes in Escherichia coli
Source: Mol Syst Biol. 2020 Mar 16;16(3):e9265. doi: 10.15252/msb.20199265 (PMC7073797; doi:10.15252/msb.20199265)
Supplement: Supplementary file 1 — Appendix [file MSB-16-e9265-s001.docx]

**Table of Contents:**

1. Appendix Figure S1 1
2. Appendix Figure S2 2
3. Appendix Figure S3 3
4. Appendix Figure S4 4
5. Appendix Figure S5 5
6. Appendix Figure S6 6
7. Appendix Figure S7 7
8. Appendix Figure S8 8
9. Appendix Table S1 9
10. Appendix Table S2 10
11. Appendix Table S2 11


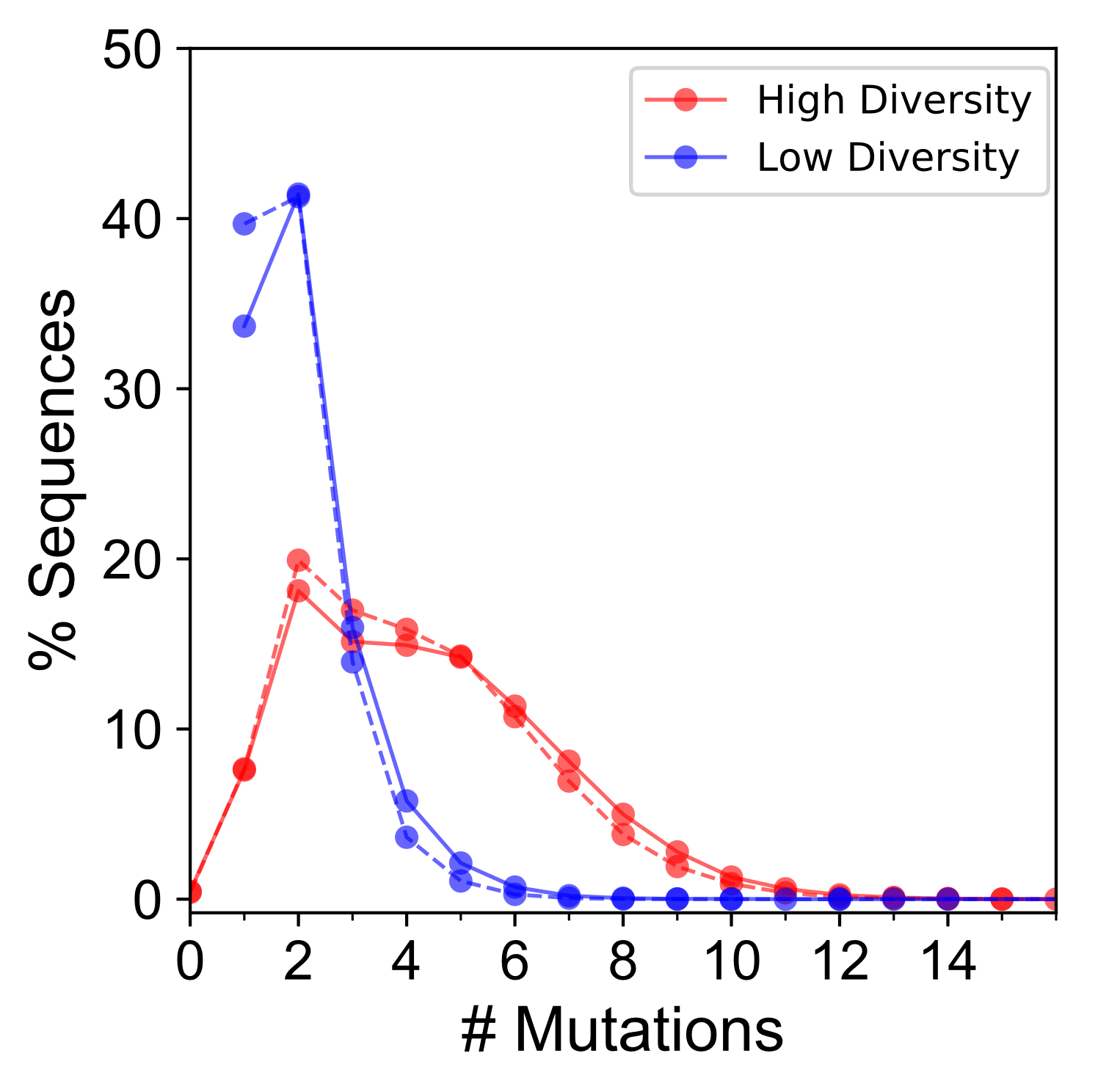


**Appendix Figure S1: Distribution of mutations in donors**

1. A comparison of percentage sequence variants categorized by the number of mutations (x-axis) between the high-diversity donor (red) and the low-diversity donor (blue)


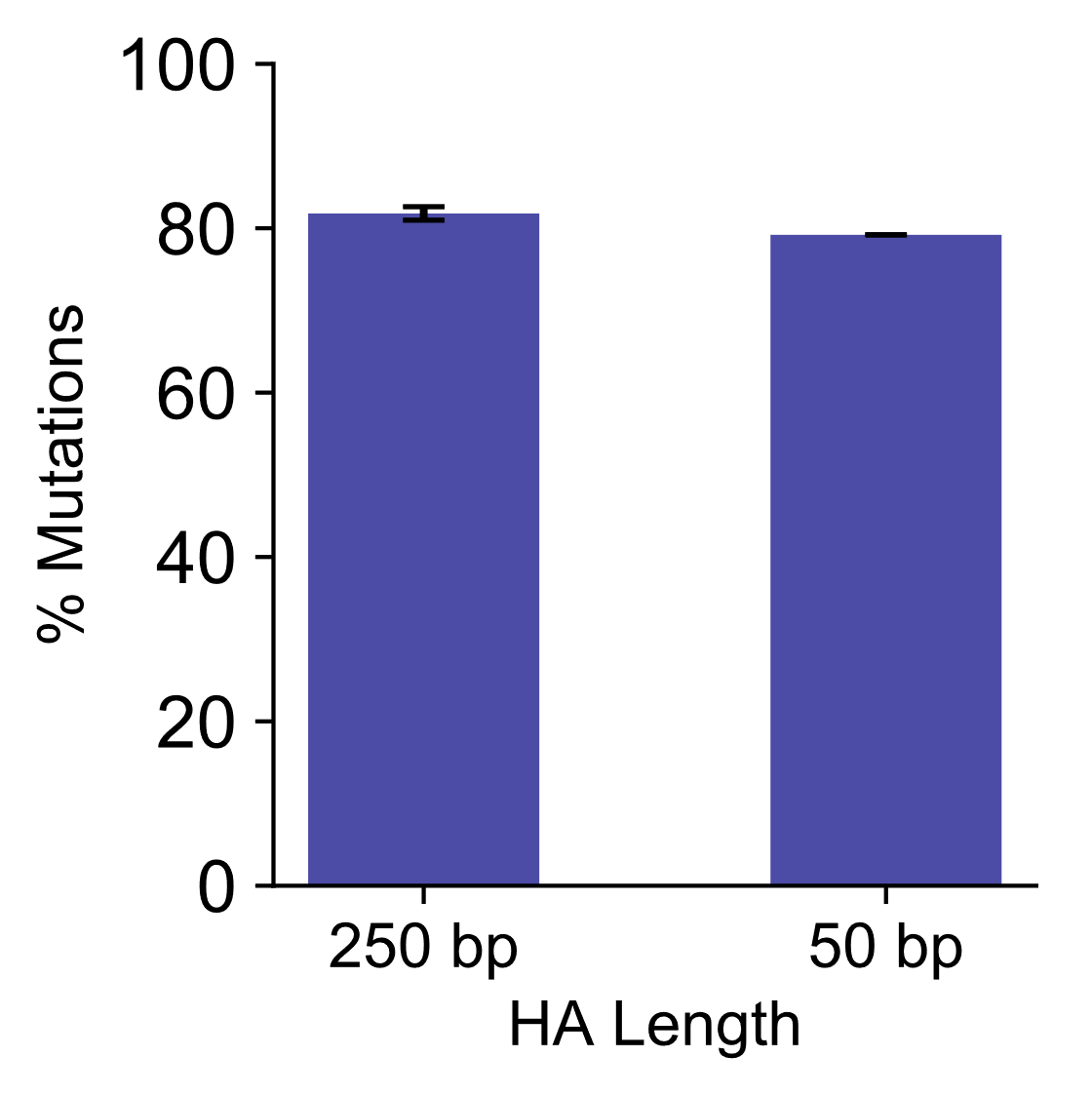


**Appendix Figure S2:** Comparison of % mutation efficiency with the high-diversity donor using pCREPE+mutL+E32K repair plasmid with repair template with end homology of lengths of 50 bp and 250 bp respectively. The mutation efficiency was determined by sequencing 25 colonies from biological replicate experiments in each case.


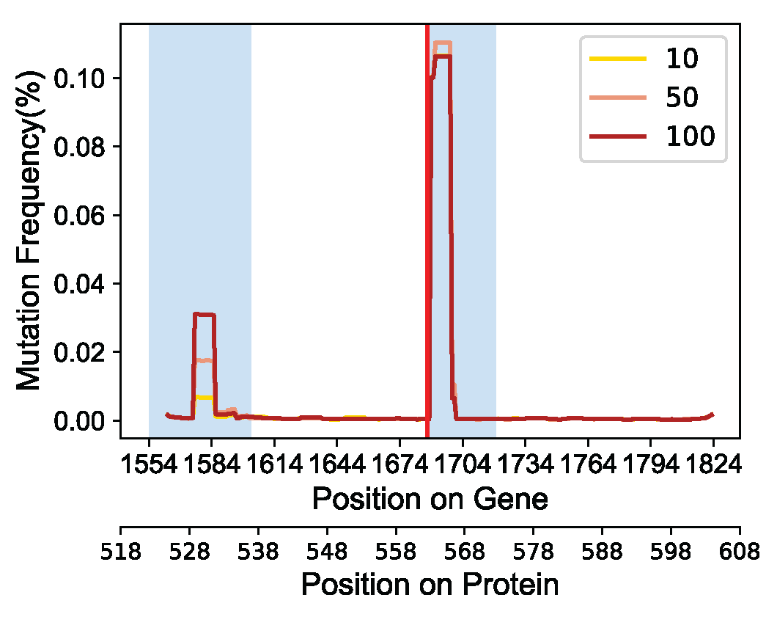


**Appendix Figure S3: Distribution of mutations after Rifampicin resistance upon targeting rpoB with the gRNA + recombination template with only the SPM**

1. The change in mutation frequency per base (%), percentage of sequences with a mutation at the position, represented as rolling mean over 10 bases along the length of the targeted region for the cells with the *rpoB* genomic error-prone PCR library at different concentrations of Rifampicin, 10 µg/mL (yellow) , 50  µg/mL (pink), and 100 µg/mL(red).


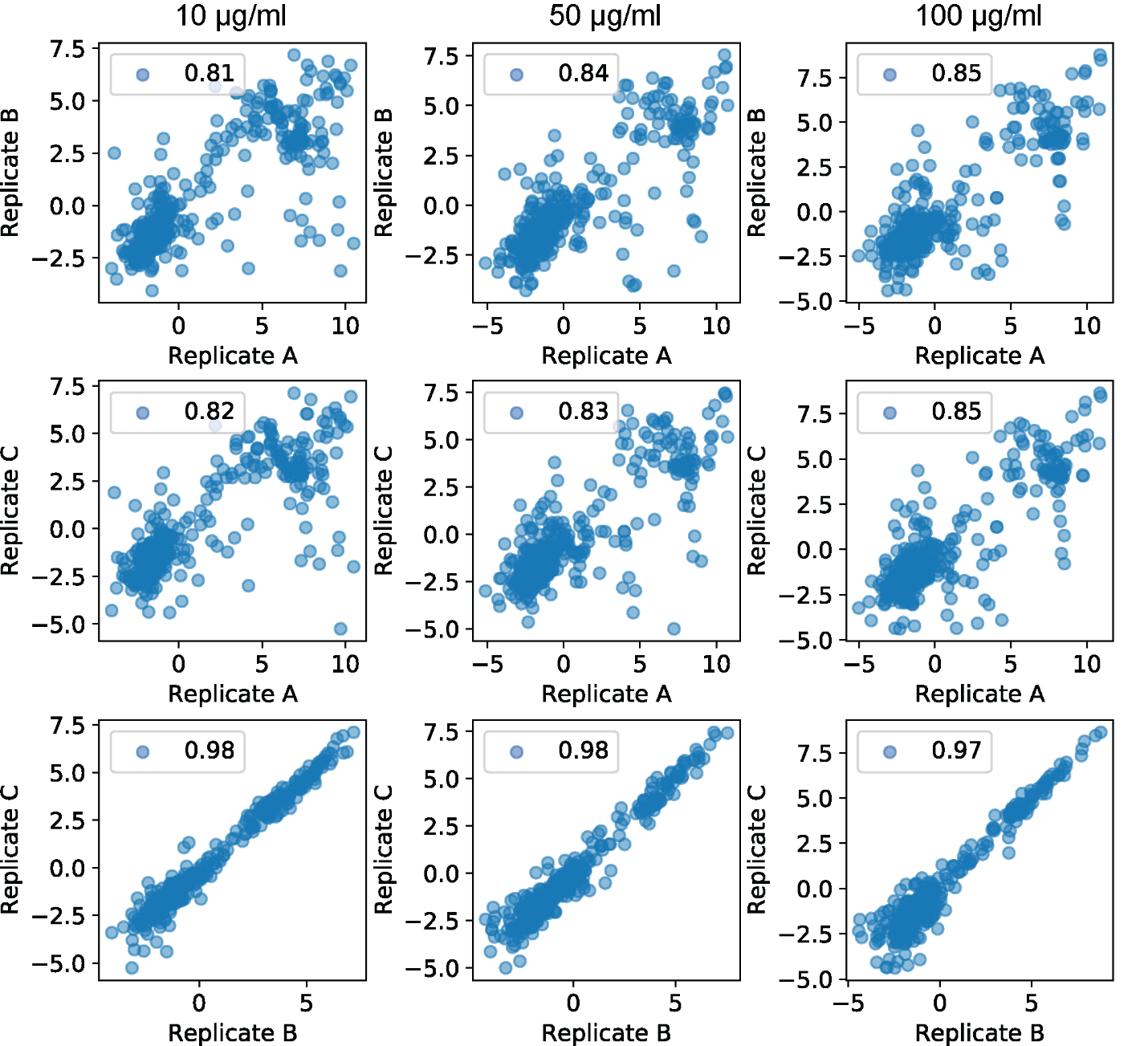


**Appendix Figure S4: Comparing fitness measures across replicates**

1. Correlation of fitness across different replicates experiments for studying Rifampicin resistance at 3 different concentration of Rifampicin. The pearson correlation coefficient is stated as legend in each box.


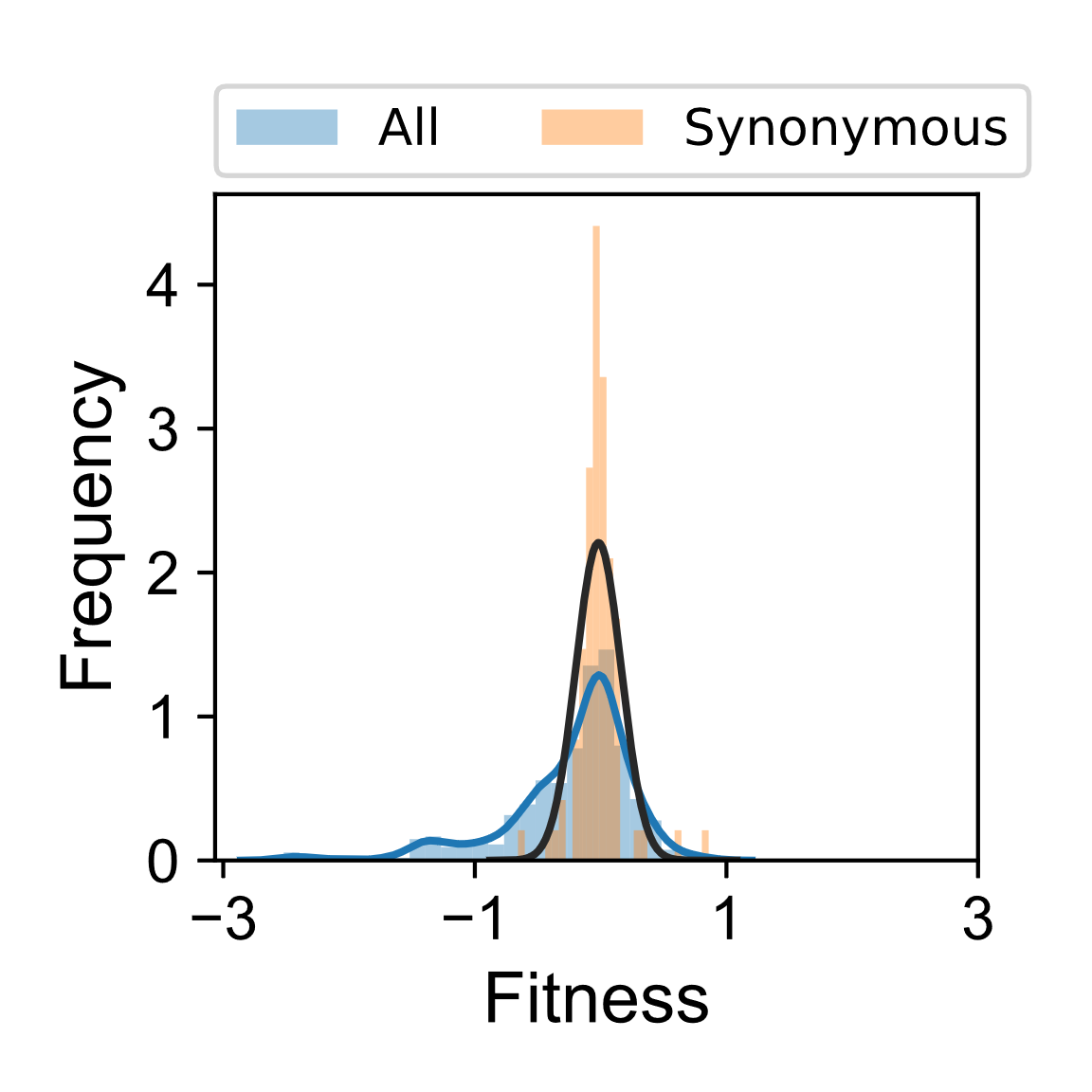


**Appendix Figure S5: Distribution of fitness effects in the absence of Rifampicin**

1. Distribution of fitness estimates for all mutations in the *rpoB* library (blue with blue line) and only synonymous mutations (salmon with the black line representing a fit for the normal distribution to estimate the mean and standard deviation of distribution of fitness effects) in the *rpoB* library in the absence of rifampicin


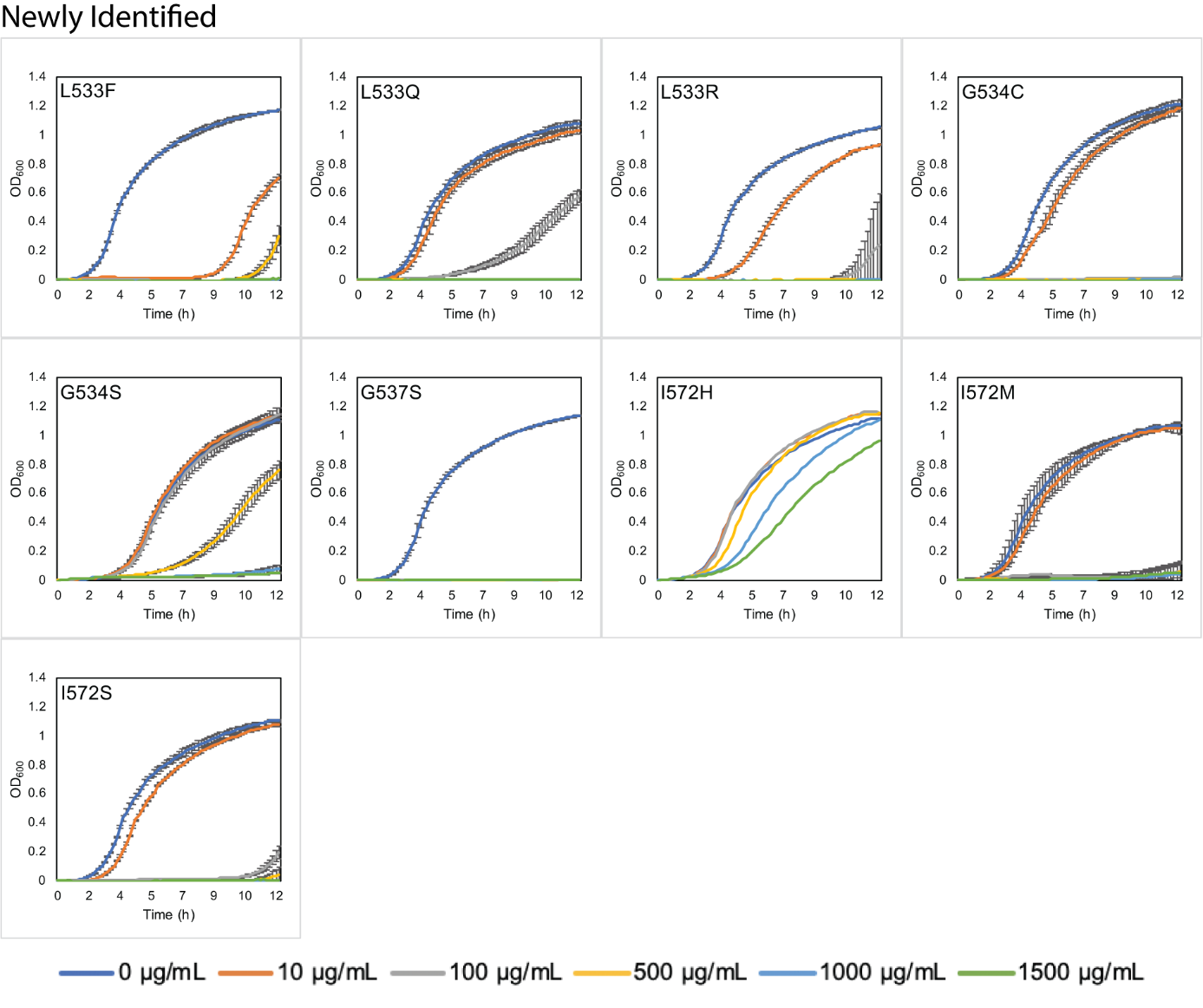


**Appendix Figure S6: Growth of newly identified mutations with Resistance to rifampicin**

1. Growth curves for the newly identified mutations at different concentrations of Rifampicin, compared to the wild-type MG1655 cells.


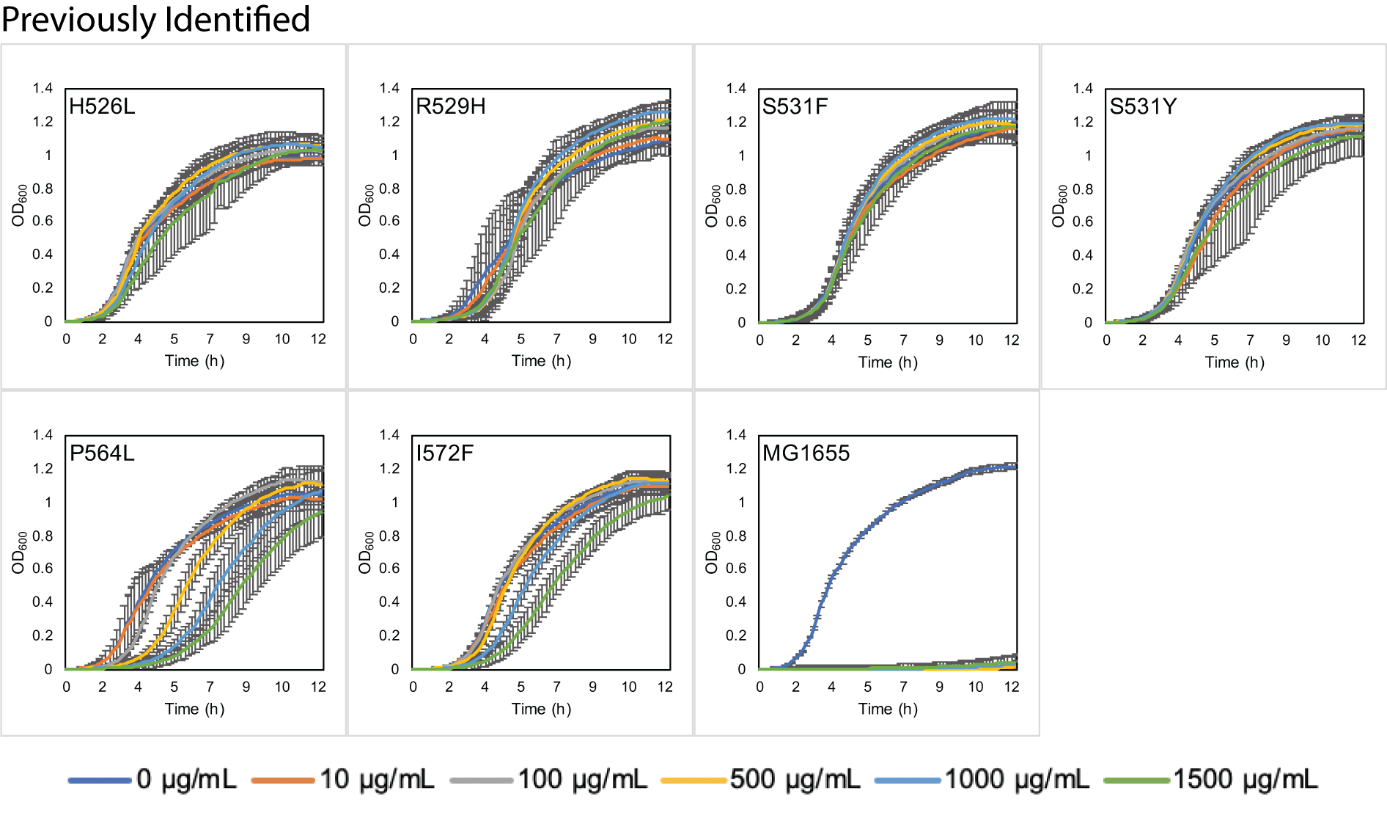


**Appendix Figure S7: Growth of previously identified mutations with Resistance to rifampicin**

1. Growth curves for the previously identified mutations at different concentrations of Rifampicin, compared to the wild-type MG1655 cells.


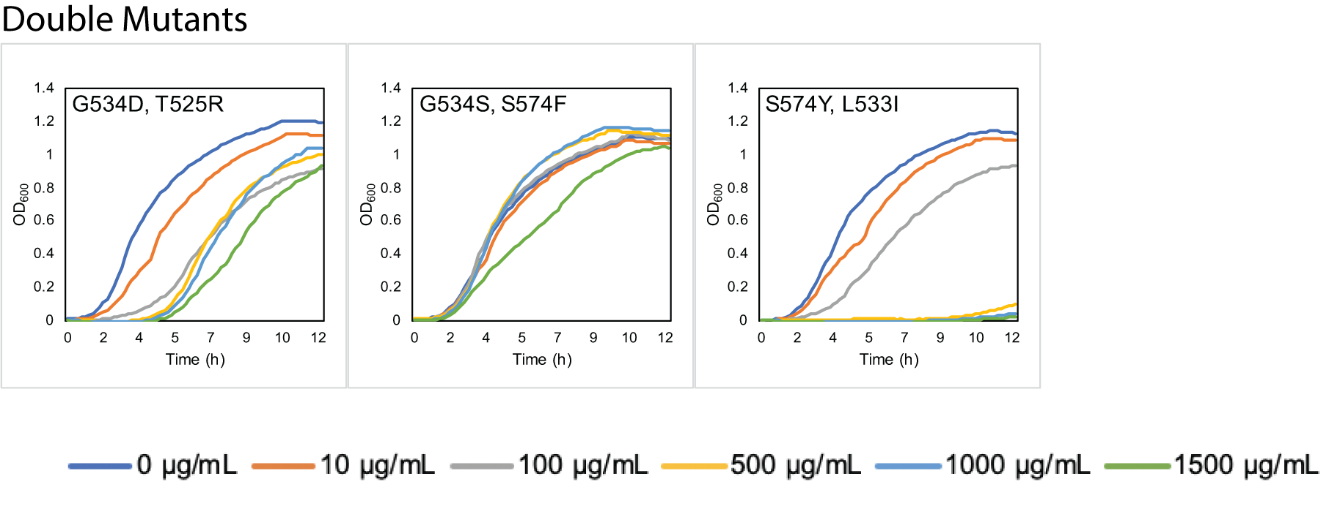


**Appendix Figure S8: Growth of double-mutants for resistance to rifampicin**

1. Growth curves for the double mutations at different concentrations of Rifampicin, compared to the wild-type MG1655 cells.

**Appendix Table S1:** List of deleterious mutations

| **Serial Number** | **Mutation** | **Position** |
| --- | --- | --- |
| 1 | R528P | 528 |
| 2 | R529P | 529 |
| 3 | R529S | 529 |
| 4 | R529L | 529 |
| 5 | F545I | 545 |
| 6 | R548L | 548 |
| 7 | D549V | 549 |
| 8 | D549Y | 549 |
| 9 | D549E | 549 |
| 10 | H551Y | 551 |
| 11 | G556V | 556 |
| 12 | G556D | 556 |
| 13 | C559R | 559 |
| 14 | I561F | 561 |
| 15 | E562V | 562 |
| 16 | E562D | 562 |
| 17 | E562G | 562 |
| 18 | T563R | 563 |
| 19 | E565K | 565 |
| 20 | N568Y | 568 |
| 21 | N568D | 568 |
| 22 | G570D | 570 |
| 23 | G570C | 570 |
| 24 | L571Q | 571 |
| 25 | S576Y | 576 |

**Appendix Table S2:** List of identified positive mutations

| **Positive Mutations** | | | | | |
| --- | --- | --- | --- | --- | --- |
| **100** | **50** | **10** | **Position** | **Known** | **RRDR** |
| H526Y | H526Y | H526Y | 526 | Yes | Yes |
|  |  | H526Q | 526 | Yes | Yes |
| H526P | H526P | H526P | 526 | Yes | Yes |
| H526L | H526L | H526L | 526 | Yes | Yes |
| H526N | H526N | H526N | 526 | Yes | Yes |
|  |  | R528L | 528 |  |  |
| R529H | R529H | R529H | 529 | Yes | Yes |
| S531Y | S531Y | S531Y | 531 | Yes | Yes |
|  |  | S531C | 531 | Yes | Yes |
| S531F | S531F | S531F | 531 | Yes | Yes |
| S531N | S531N | S531N | 531 |  | Yes |
|  |  | A532P | 532 |  | Yes |
|  |  | L533F | 533 |  | Yes |
|  |  | L533H | 533 | Yes | Yes |
|  |  | L533I | 533 |  | Yes |
| L533P | L533P | L533P | 533 | Yes | Yes |
|  |  | L533V | 533 |  | Yes |
|  |  | G534V | 534 |  | Yes |
|  |  | G534S | 534 |  | Yes |
|  |  | G534R | 534 |  | Yes |
|  |  | G534D | 534 | Yes | Yes |
|  |  | G534C | 534 |  | Yes |
|  |  | G537A | 537 |  |  |
|  |  | G537S | 537 |  |  |
| L538P | L538P | L538P | 538 | Yes | Yes |
|  | G544V |  | 544 |  | Yes |
|  |  | P564R | 564 |  | Yes |
| P564L | P564L | P564L | 564 | Yes | Yes |
|  |  | I572T | 572 |  | Yes |
|  |  | I572S | 572 |  | Yes |
|  |  | I572M | 572 |  | Yes |
| I572L | I572L | I572L | 572 |  | Yes |
| I572F | I572F | I572F | 572 | Yes | Yes |
| I572Y | I572Y | I572Y | 572 |  | Yes |
|  |  | S574Y | 574 |  | Yes |
|  |  | S574F | 574 | Yes | Yes |
| T581A | T581A | T581A | 581 |  |  |
| T595S |  |  | 595 |  |  |

**Appendix Table S3:** List of primers

| **Name** | **Sequence** | **Notes** |
| --- | --- | --- |
| **F_crpW1** | cagaaaagttaacccttcgacccac | Genome ampilifciation of  crp window |
| **R_crpW1** | gatcttccagcatcttcagaatgcg | Genome ampilifciation of  crp window |
| **F_pSAH_crp-W1** | cgtgaaaccgtgggacgcattctgaagatgctggaagatcAGCGAGAGACGGACACGAAC | Backbone ampilifciation to  clone crp window |
| **R_pSAH_crp-W1** | gcaagcgcgagtgaagtgggtcgaagggttaacttttctgGGACAGAGACGGCTGAGGTG | Backbone ampilifciation to  clone crp window |
| **F_mreB-W1** | tgttgaaaaaatttcgtggcatgttttcc | Genome ampilifciation of  mreB window |
| **R_mreB-W1** | ttccagtgcaaccattaccgc | Genome ampilifciation of  mreB window |
| **F_pSAH_mreB-W1** | gctgaccggtattgtgagcgcggtaatggttgcactggaaAGCGAGAGACGGACACGAACC | Backbone ampilifciation to  clone mreB window |
| **R_pSAH_mreB-W1** | gacaagtcattggaaaacatgccacgaaattttttcaacaGGACAGAGACGGCTGAGGTG | Backbone ampilifciation to  clone mreB window |
| **F_rpoB_W1** | gtatcctgagcaaagacgacatcattg | Genome ampilifciation of  rpoB window |
| **R_rpoB_W1** | caacagcacgttccataccagtac | Genome ampilifciation of  rpoB window |
| **F_pSAH_rpoB_W1** | gataagccgctggttggtactggtatggaacgtgctgttgAGCGAGAGACGGACACGAAC | Backbone ampilifciation to  clone rpoB window |
| **R_pSAH_rpoB_W1** | tttttcataacatcaatgatgtcgtctttgctcaggatacGGACAGAGACGGCTGAGGTG | Backbone ampilifciation to  clone rpoB window |
| **crp1_mut_f** | gtgcttggcaaaccgcaaac | Error-prone PCR of  crp window |
| **crp1_mut_r** | ggttttcgcacgtacccatg | Error-prone PCR of  crp window |
| **mreB1_mut_f** | cggcgttatcgccgacttct | Error-prone PCR of  mreB window |
| **mreB1_mut_r** | ccaccgatatcaaccaccat | Error-prone PCR of  mreB window |
| **rpoB1_mut_f** | caacccgctgtctgagatta | Error-prone PCR of  rpoB window |
| **rpoB1_mut_r** | gcagacaggtagtgaatttc | Error-prone PCR of  rpoB window |
| **crp1_bb_r** | agtcgggtctgtttgcggtt | Backbone ampilifciation to  clone crp error-prone library |
| **crp1_bb_f** | gaacgtagcgcatgggtacg | Backbone ampilifciation to  clone crp error-prone library |
| **mreB1_bb_r** | tcagtcacgaagaagtcggc | Backbone ampilifciation to  clone mreB error-prone library |
| **mreB1_bb_f** | gaccggttctatggtggttg | Backbone ampilifciation to  clone mreB error-prone library |
| **rpoB1_bb_f** | tgtaactgacgaaattcact | Backbone ampilifciation to  clone rpoB error-prone library |
| **rpoB2_bb_r** | ccttcttcatccaagttgga | Backbone ampilifciation to  clone rpoB error-prone library |
| **galK_nextgenseq_for** | TCGTCGGCAGCGTCAGATGTGTATAAGAGACAGNNNNNNAGTTTTCCCTCGATGCGCCC | Next-gen sequencing of *galK* library |
| **galK_nextgenseq_rev** | GTCTCGTGGGCTCGGAGATGTGTATAAGAGACAGNNNNNNTGTTGATGATGACGACAGCCA | Next-gen sequencing of *galK* library |
| **crp_nextgenseq_for** | TCGTCGGCAGCGTCAGATGTGTATAAGAGACAGNNNNNNgtgcttggcaaaccgcaaac | Next-gen sequencing of *crp* library |
| **crp_nextgenseq_rev** | GTCTCGTGGGCTCGGAGATGTGTATAAGAGACAGNNNNNNggttttcgcacgtacccatg | Next-gen sequencing of *crp* library |
| **mreB_nextgenseq_for** | TCGTCGGCAGCGTCAGATGTGTATAAGAGACAGNNNNNNcggcgttatcgccgacttct | Next-gen sequencing of *mreB* library |
| **mreB_nextgenseq_rev** | GTCTCGTGGGCTCGGAGATGTGTATAAGAGACAGNNNNNNccaccgatatcaaccaccat | Next-gen sequencing of *mreB* library |
| **rpoB_nextgenseq_for** | TCGTCGGCAGCGTCAGATGTGTATAAGAGACAGNNNNNNcaacccgctgtctgagatta | Next-gen sequencing of *rpoB* library |
| **rpoB_nextgenseq_rev** | GTCTCGTGGGCTCGGAGATGTGTATAAGAGACAGNNNNNNgcagacaggtagtgaatttc | Next-gen sequencing of *rpoB* library |
| **galK gRNA** | atgataaagctgctgcaata | gRNA spacer to manipulate *galK* |
| **crp gRNA** | ctacatcgttaaaggctctg | gRNA spacer to manipulate *crp* |
| **mreB gRNA** | gcgcggattcacgaattgcg | gRNA spacer to manipulate *mreB* |
| **rpoB gRNA** | accgatgttcggaccttcagg | gRNA spacer to manipulate *rpoB* |
| **galK_HA** | GGGAGGCTCTTCTTTTTCATATTATTGAGCATTTATCAGGGTTATTGTCTCATGAGCGGATACATATTTGAATGTATTTAGAAAAATAAACAAATAGGGGTTCCGCGCACATTTCCCCGAAAAGTGCCACCTGACGTCTAAGAAACCATTATTATCATGACATTAACCTATAAAAATAGGCGTATCACGAGGCAGAATTTCAGATAAAAAAAATCCTTAGCTTTCGCTAAGGATGATTTCTGGAATTCTAAAGATCTCCGCAGGGTGCCGGGTTAAGTTCTTCCGCTTCACTGGAAGTCGCGGTCGGAACGGTATTGCAGCAGCTTTAACATCTGCCGCTGGACGGCGCACAAATCGCGCTTAACGGTCAGGAAGCAGATC | Recombination template to  introduce SPM in *galK* |
| **Crp_1-1_tgg_ttg_HA** | TAGCTTTCGCTAAGGATGATatggtgcttggcaaaccgcaaacagacccgactctcgaatggttcttgtctcattgccacattcataagtacccatccaagagcacgcttattcaccagggtgaaaaagcggaaacgctgtactacatcgttaaaggctctgttgcagtgctgatcaaagacgaagagggtaaagaaatgatcctctcctatctgaatcagggtgattttattggcgaactgggcctgtttgaagagggccaggaacgtagcgcatgggtacgtgcgaaaaccgcctgtgaagtggctgaaatttcgtacttgacagctagctcagtcct | Recombination template to  introduce SPM in *crp* |
| **mreB_1-1_R123_CGC_CGT** | TAGCTTTCGCTAAGGATGATctccagcacttcatcaaacaagtgcacagcaacagctttatgcgtccaagcccgcgcgttctggttt  gtgtgccggttggcgcgacccaggttgaacgTcgcgcaattcgtgaatccgcgcagggcgctggtgcccgtgaagtcttcctgattgaagaaccgatggctgccgcaattggtgctggcctgccggtttctttgacagctagctcagtcct | Recombination template to  introduce SPM in *mreB* |
| **rpoB_1-1_ACC__ACG** | TAGCTTTCGCTAAGGATGATatctccgcactcggcccaggcggtctgacccgtgaacgtgcaggcttcgaagttcgagacgtacacccgactcactacggtcgcgtatgtccaatcgaaacGcctgaaggtccgaacatcggtctgatcaactctctgtccgtgtacgcacagactaacgaatacggcttccttgagactccgtatcgtaaagtgaccgacttgacagctagctcagtcct | Recombination template to  introduce SPM in *rpoB* |
