# Supplementary material for: CRISPR/Cas9 recombineering‐mediated deep mutational scanning of essential genes in Escherichia coli
Source: Mol Syst Biol. 2020 Mar 16;16(3):e9265. doi: 10.15252/msb.20199265 (PMC7073797; doi:10.15252/msb.20199265)
Supplement: Supplementary file 2 — Expanded View Figures PDF [file MSB-16-e9265-s002.pdf]

Expanded View Figures

Figure EV1. Impact of donor diversity and homology arm length on mutation efficiency using a low-diversity donor.

A Comparison of mutation efficiency between the high- and low-diversity donors before (black) and after (blue) integration on the genome. The lighter colors represent the fraction of sequences with only the SPM. Each value represents the mean, and error bars represent the standard deviation for two biological replicates of deep sequencing data.

B A comparison of percentage sequence variants categorized by the number of mutations (*x*-axis) between the low-diversity donor before (red) and after (blue) integration on the genome. #Mutations = 1 corresponds to sequences with only the SPM. The experiments were performed in biological replicates. The trends for the replicates are represented by solid (—) and dashed (--) lines, respectively.

C Change in mutation frequency per base (%) and percentage of sequences with a mutation at the position, using the low-diversity donor, are represented as the rolling mean over 10 bases versus the distance from the PAM measured in base pairs.

D Comparison of % mutation efficiency determined by deep sequencing the genome after integrating the low-diversity donor using end homology of lengths of 50, 150, and 250 bp. Each value represents the mean, and error bars represent the standard deviation for two biological replicates.

E Comparison of the change in mutation frequency per base (%) and percentage of sequences with a mutation at each position, using the high-diversity donor, are represented as the rolling mean over 10 bases versus the distance from the PAM (base pairs) for recombination of the low-diversity donor using 50- and 250-bp end homology.

F A comparison of percentage sequence variants categorized by the number of mutations (*x*-axis) for the low-diversity donor before (red) and after genome integration using 50-bp (green)- and 250-bp (blue)-long end homology. The trends for the replicates are represented by solid (—) and dashed (--) lines, respectively.

© 2020 The Authors

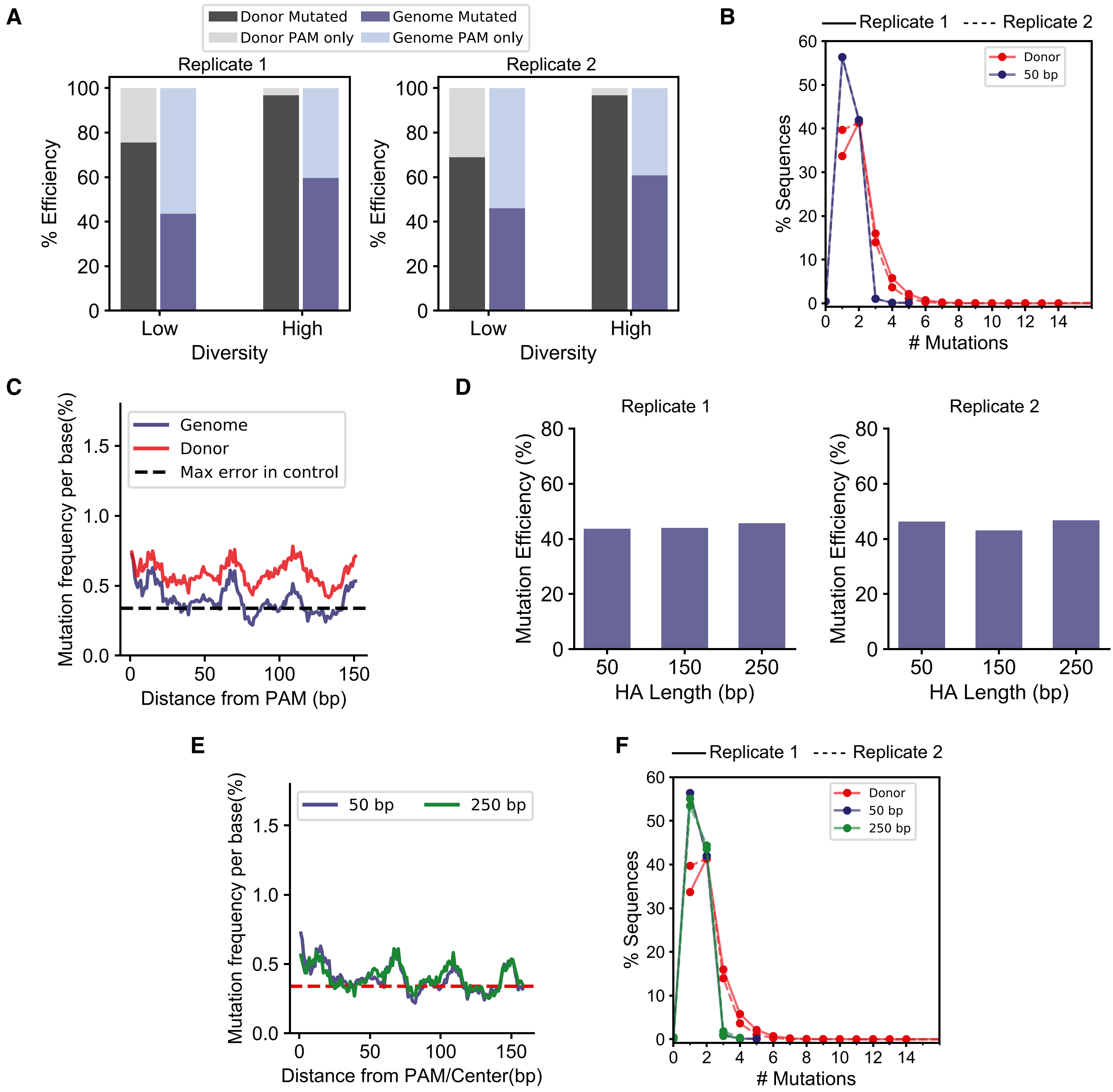

Figure EV1.

© 2020 The Authors

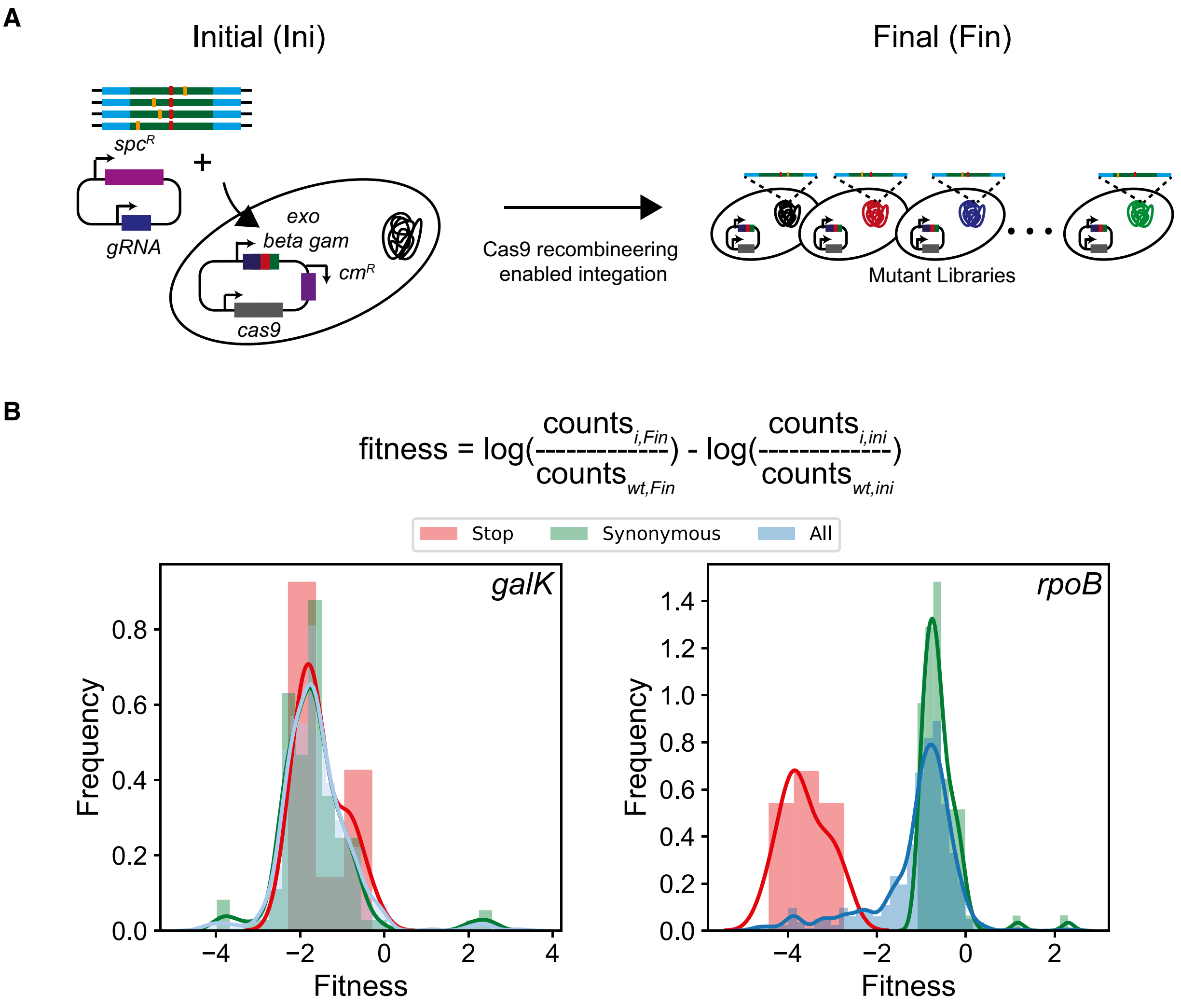

Figure EV2. Identification of deleterious substitutions using CREPE.

A We compared the frequency of variants efficiency before (Initial, ini) and after (Final, fin) integration on the genome.

B Distribution of fitness estimates for integration of the donor (using the fitness equation as highlighted in the text) for the *galK* (left), and *rpoB* (right) for non-synonymous (blue with blue line), synonymous mutations (green with green line), and stop codon (red with red line) substitutions.

© 2020 The Authors

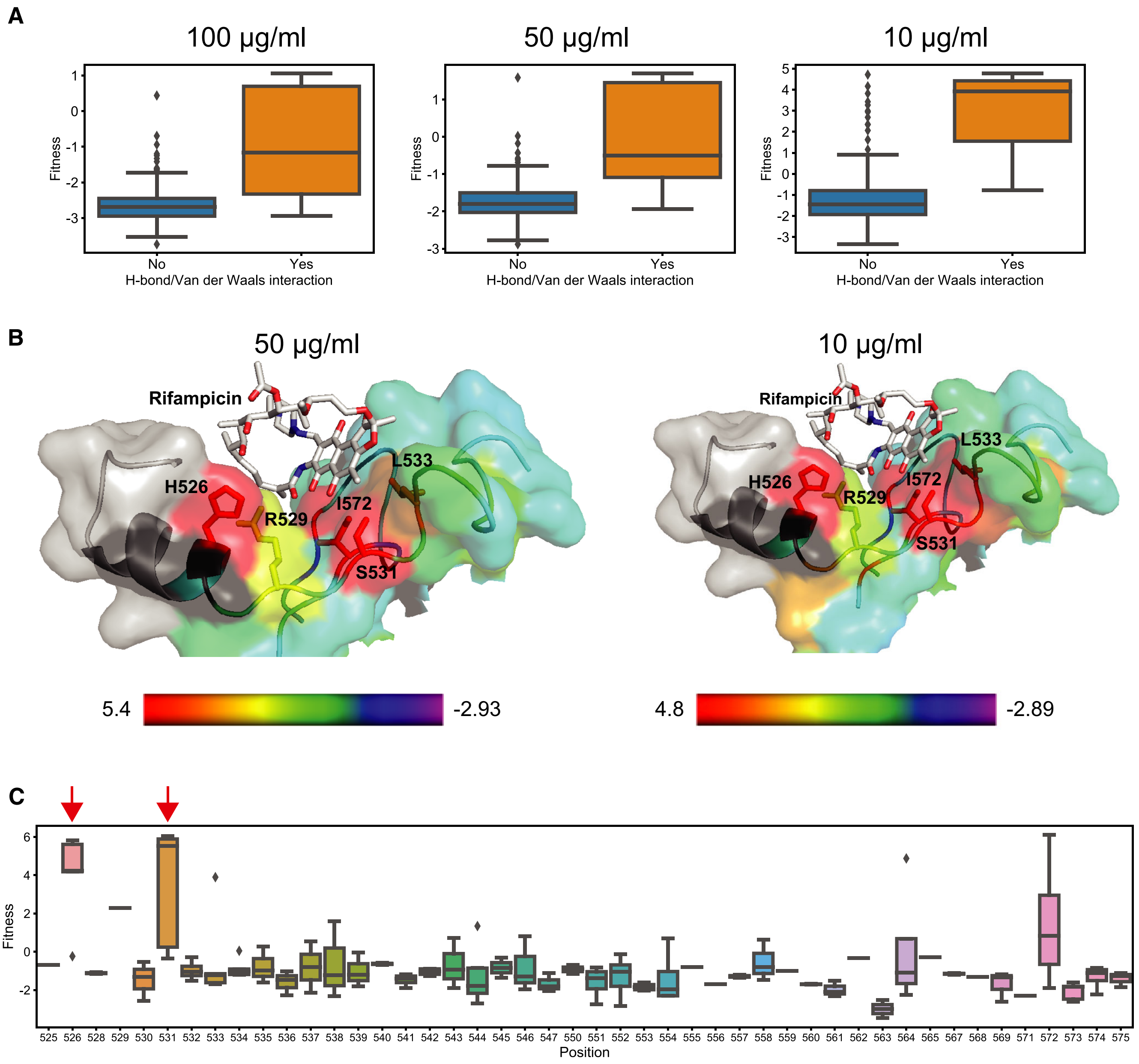

Figure EV3. Evaluation of mutation fitness scores based on biochemical and structural properties.

A Each plot compares the distribution of fitness between residues whose side chains that make hydrogen bonds or van der Waals interactions with rifampicin (orange) and residues that do not interact with rifampicin (blue) at 100 µg/ml (left), 50 µg/ml (middle), and 10 µg/ml (right) of rifampicin. Within the box, the central line represents the median, the bottom and top edges of the box represent the 25 and 75% quartiles, the bottom and top whiskers represent 9 and 91% percentiles, and the dots represent outliers.

B The structure (cartoon + surface) represents rifampicin (white sticks)-binding pocket. The residues are colored using a heat map (range below the structure) demonstrating the maximum fitness at each residue. Residues with sticks form H bonds or van der Waals interactions with rifampicin (Campbell *et al*, 2001). The fitness scores were evaluated at 50 µg/ml rifampicin (left) and 10 µg/ml rifampicin (right). The structure with PDB code 5UAC was used for representation (Molodtsov *et al*, 2017).

C Each box plot represents the distribution of fitness effects for mutations evaluated at each amino acid of the targeted RpoB region in RRDR I (525–538) and RRDR II (562–575), and the residues between the RRDRs at 100 µg/ml rifampicin. The red arrows represent the residues in which the mutations account for over 90% of clinically known resistance-conferring mutations. Within the box, the central line represents the median, the bottom and top edges of the box represent the 25 and 75% quartiles, the bottom and top whiskers represent 9 and 91% percentiles, and the dots represent outliers.

 © 2020 The Authors

Figure EV4. Comparison of fitness distributions between low and high rifampicin concentrations.

A Correlation of fitness estimated for the same mutant at 50 μg/ml rifampicin (*x*-axis) and 100 μg/ml rifampicin (*y*-axis) (left), and 10 μg/ml rifampicin (*x*-axis), and 50 μg/ml rifampicin (*y*-axis) (right). The orange and blue regions represent the window within 2.96 fitness standard deviations (on each side) around the mean fitness of synonymous mutations at 10 and 100 μg/ml respectively.

B In each structure, rifampicin (white sticks colored by atoms) is bound to rpoB (blue cartoon). The amino acid side chain in sticks represents the S531 residue in the wild-type sequence (S531S, left), and when it is mutated to high-resistance residue (S531L, middle) and low-resistance residue (S531A, right). The structure with PDB code 5UAC was used for representation (Molodtsov *et al*, 2017).

C In each structure, rifampicin (white sticks colored by atoms) is bound to rpoB (blue cartoon). The amino acid side chain in sticks represents the I572 residue in the wild-type sequence (I572I, left), and when it is mutated to high-resistance residue (I572F and I572L, middle) and low-resistance residue (I572S and I572T, right). The structure with PDB code 5UAC was used for representation (Molodtsov *et al*, 2017).

© 2020 The Authors

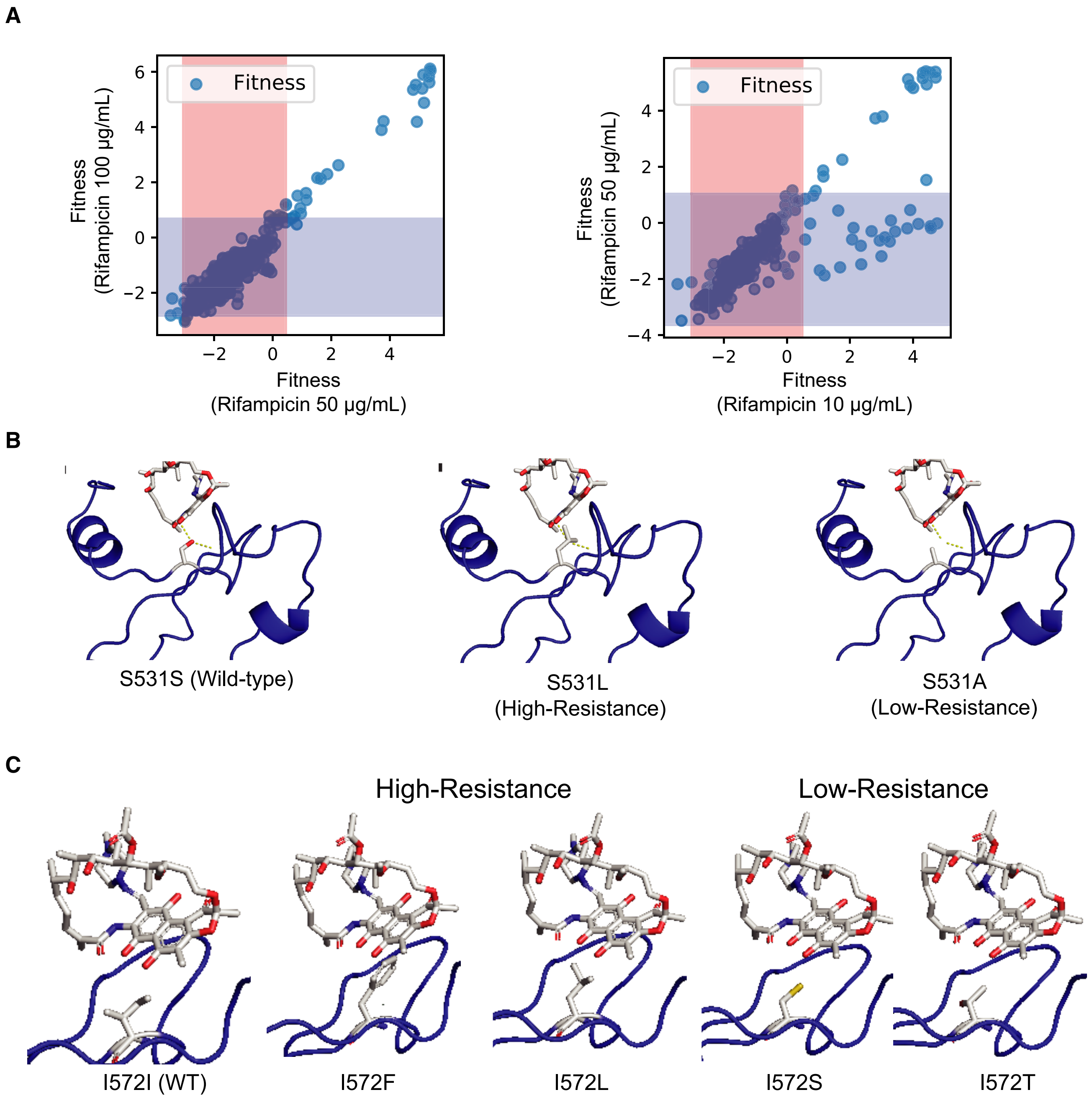

Figure EV4.

 © 2020 The Authors

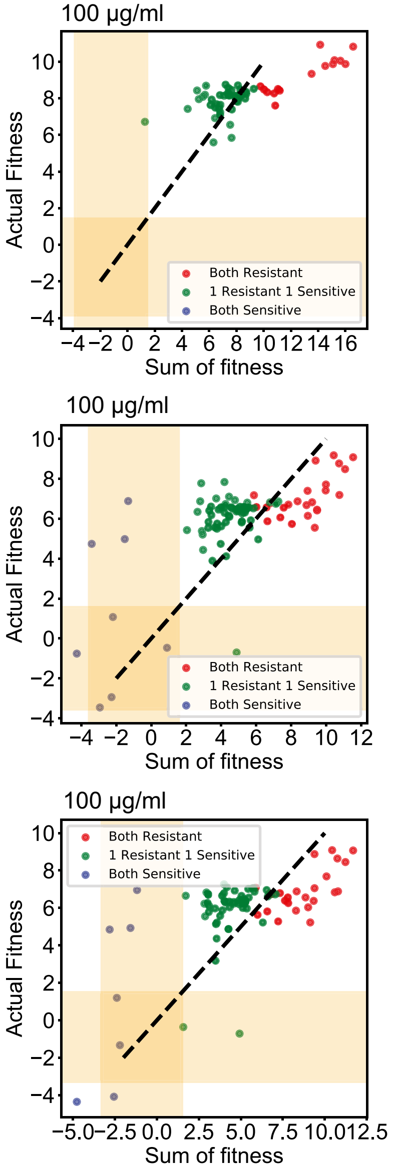

Figure EV5. Epistasis across replicates and between concentrations.

Comparison of actual fitness determined by CREPE and predicted fitness determined as the sum of fitness for individual mutations in the double mutants for resistance to different concentrations of rifampicin across the 3 biological replicates. The double mutants were categorized as either the combination of 2 resistant mutations (red), 1 resistant and 1 sensitive mutant (green), or 2 sensitive mutations (blue).

© 2020 The Authors
